# Relaxant effects of a hydroalcoholic extract of *Ruta graveolens* on isolated rat tracheal rings

**DOI:** 10.1186/s40659-015-0017-8

**Published:** 2015-06-05

**Authors:** Luis Águila, Jenny Ruedlinger, Karina Mansilla, José Ordenes, Raúl Salvatici, Rui Ribeiro de Campos, Fernando Romero

**Affiliations:** Center of Neurosciences and Peptides Biology (CEBIOR-BIOREN), Faculty of Medicine, University of La Frontera, Temuco, Chile; Center of Genetic and Immunologic Studies (CEGIN), Faculty of Medicine, University of La Frontera, Temuco, Chile; School of Veterinary Medicine, Unit of Nutrition, and Animal Production, Faculty of Natural Resources, and Veterinary Medicine, Santo Tomás University, Temuco, Chile; Department of Physiology, Cardiovascular Division, Federal University of Sao Paulo, São Paulo, Brazil

**Keywords:** Ruta graveolens, Trachea-relaxant, Plant extract

## Abstract

**Background:**

*Ruta graveolens L.* (*R. graveolens*) is a medicinal plant employed in non-traditional medicines that has various therapeutic properties, including anthelmintic, and vasodilatory actions, among others. We evaluated the trachea-relaxant effects of hydroalcoholic extract of *R. graveolens* against potassium chloride (KCl)- and carbachol-induced contraction of rat tracheal rings in an isolated organ bath.

**Results:**

The results showed that the airway smooth muscle contraction induced by the depolarizing agent (KCl) and cholinergic agonist (carbachol) was markedly reduced by *R. graveolens* in a concentration-dependent manner, with maximum values of 109 ± 7.9 % and 118 ± 2.6 %, respectively (changes in tension expressed as positive percentages of change in proportion to maximum contraction), at the concentration of 45 μg/mL (half-maximal inhibitory concentration IC_50_: 35.5 μg/mL and 27.8 μg/mL for KCl- and carbachol-induced contraction, respectively). Additionally, the presence of *R. graveolens* produced rightward parallel displacement of carbachol dose–response curves and reduced over 35 % of the maximum smooth muscle contraction.

**Conclusions:**

The hydroalcoholic extract of *R. graveolens* exhibited relaxant activity on rat tracheal rings. The results suggest that the trachea-relaxant effect is mediated by a non-competitive antagonistic mechanism. More detailed studies are needed to identify the target of the inhibition, and to determine more precisely the pharmacological mechanisms involved in the observed biological effects.

## Background

Asthma is an airway allergic inflammatory disease characterized by bronchospasms, intermittent chronic inflammation, and airway remodeling [1, 2]. Meanwhile, chronic obstructive pulmonary disease (COPD) is a progressive pathology characterized by increased airflow restriction [3, 4]. In recent years, efforts to develop more beneficial and safer therapies for these conditions have increased, focusing on prevention rather than treatment of the active diseases [5, 6]. The current challenge in research is to identify the molecular mediators and mechanisms involved in the pathophysiology of such diseases [7, 8]. Thus, several pharmacological studies of natural products capable of relaxing the airway smooth muscle to improve the airflow have been ongoing, and are considered as alternative treatments.

Several plants of the *Rutaceae* family are used in non-traditional medicines around the world. The most common plant is *Ruta graveolens L* (*R. graveolens*), popularly known as “Ruta”. This plant was brought to Chile from southern Europe. In folk medicines, it has mainly been used for menstrual problems, respiratory diseases, and gastrointestinal disorders [9, 10]. In several scientific reports, *R. graveolens* has been described to have hypotensive [11], spasmolytic [12], anti-inflammatory [13], sperm motility-inhibitory [14], algaecidal and antifungal [15], and antimicrobial [16] properties, and even to act as an anti-carcinogenic agent [17] and antioxidant [18–20].

Nevertheless, despite the many studies carried out on the biological effects of *R. graveolens*, there are no scientific data about its effects on airway contractility. Thus, as part of a research program developed at the Universidad de La Frontera to characterize the bioactive actions of *R. graveolens*, the aim of this study was to perform phytochemical screening of a hydroalcoholic extract of this plant and to evaluate its trachea-relaxant potential on agonist-induced rat tracheal ring contraction.

## Results

### Chromatogram analysis and identification of signals

Our phytochemical screening of the hydroalcoholic extract revealed the presence of secondary metabolites with potential biological effects (quercetin, rutin, and psoralen) (Table 1). Thus, this ethanolic extract was selected for further study.

**Table 1:** Signal identification

Retention time (min)	Q1 Precursor ions	Q3 Fragment Ions	Compounds
19.55	187	131/115	Psoralen
19.64	303	229/153	Quercetin
17.70	611	303/465	Rutin

Identification of the compounds in each sample of the *R. graveolens* extract, analyzed by comparisons with standards in both retention times and fragment settings/specific precursors

### Airway smooth muscle contraction by carbachol: determination of EC_50_

**Fig. 1:**
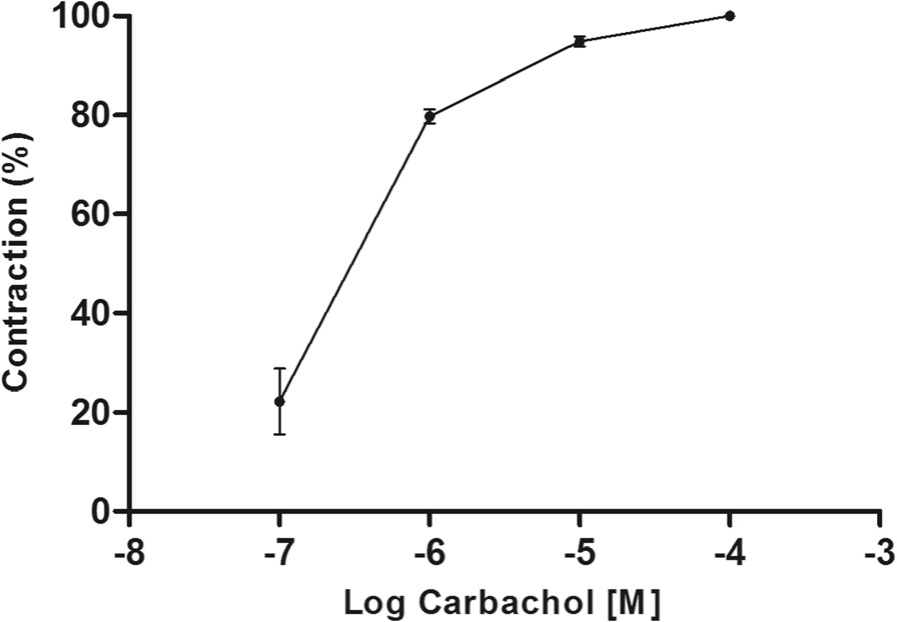
Dose–response curve of tracheal smooth muscle contraction induced by carbachol (0.1, 1.0, 10, and 100 μM). Symbols and vertical bars represent means and SEM (n = 3 biological replicates)

Increasing concentrations of carbachol (0.1–100 μM) caused concentration-dependent contraction in tracheal preparations with an EC_50_ value of 0.2 μM, reflecting the maximum contraction obtained with 100 μM carbachol (Fig. 1).

### R. graveolens extract induces relaxation of contracted rat tracheal rings to a similar level to aminophylline

**Fig. 2:**
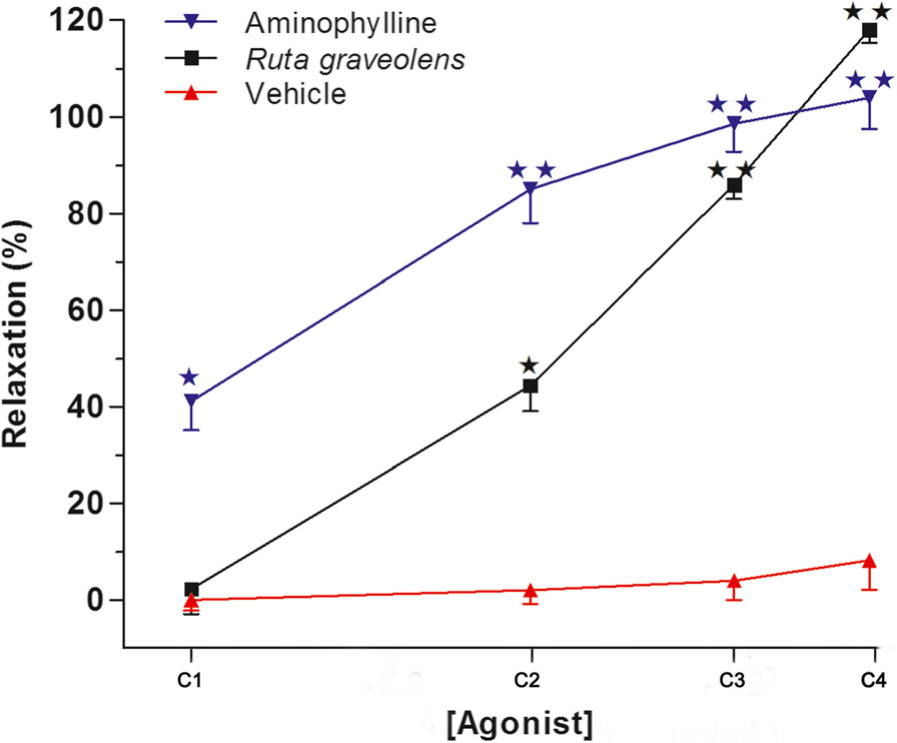
Effects of different concentrations of the *R. graveolens* extract (5–45 μg/mL) on carbachol (1 μM) pre-contracted rat tracheal rings. Concentrations of *R. graveolens*: C1, 5 μg/mL; C2, 15 μg/mL; C3, 30 μg/mL; C4, 45 μg/mL. Concentrations of aminophylline: C1, 0.2 mM; C2, 0.4 mM; C3, 0.8 mM; C4, 1.0 mM. Symbols and vertical bars represent means and SEM (n = 5 biological replicates). Data with different superscript symbols differ significantly among groups: *p < 0.05; **p < 0.01

**Fig. 3:**
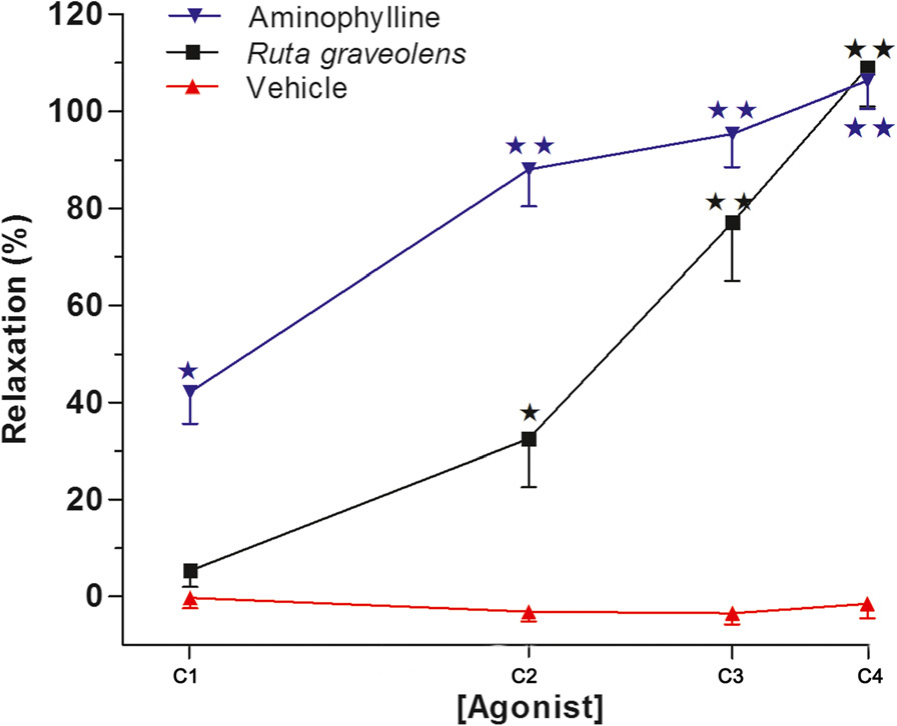
Effects of different concentrations of the *R. graveolens* extract (5–45 μg/mL) on KCl (80 mM) pre-contracted rat tracheal rings. Concentrations of *R. graveolens*: C1, 5 μg/mL; C2, 15 μg/mL; C3, 30 μg/mL; C4, 45 μg/mL. Concentrations of aminophylline: C1, 0.2 mM; C2, 0.4 mM; C3, 0.8 mM; C4, 1.0 mM. Symbols and vertical bars represent means and SEM (n = 5 biological replicates). Data with different superscript symbols differ significantly among groups: *p < 0.05; **p < 0.01

In the tracheal ring preparations, increasing concentrations of the *R. graveolens* extract (5, 15, 30, and 45 μg/mL) significantly reduced (p < 0.001) the muscle tension induced by carbachol in a concentration-dependent manner, with the maximum value of 118 ± 2.6 % (n = 5) obtained at a concentration of 45 μg/mL (EC50: 27.8 μg/mL) (Fig. 2). Meanwhile, in potassium chloride (KCl) pre-contracted tracheal rings, the muscle tension was reduced by the *R. graveolens* extract (5–45 μg/mL) in a concentration-dependent manner, with the maximum value of 109 ± 7.9 % obtained at a concentration of 45μg/mL (EC_50_: 35.5 μg/mL) (Fig. 3). The plant extract achieved relaxant effects at a similar level to those of aminophylline. However, the relaxant effects of *R. graveolens* at 5 and 15 μg/mL were significantly lower than those of 0.2 and 0.4 mM aminophylline (Figs. 2 and 3). Aminophylline (0.2, 0.4, 0.8, and 1.0 mM) significantly decreased (p < 0.001) the muscle tension induced by carbachol and KCl in a concentration-dependent manner, with maximum values of 104 ± 6.4 % and 106 ± 5.7 %, respectively (EC_50_: 6.3 μM and 6.0 μM for carbachol and KCl, respectively) (Figs. 2 and 3).

### Antagonist-like effect of the R. graveolens extract against carbachol-induced tension

**Fig. 4:**
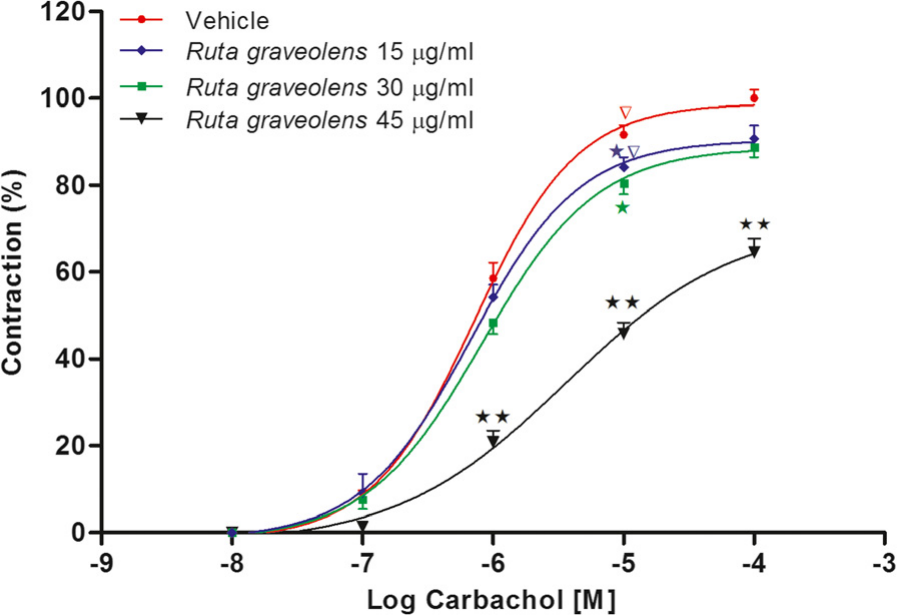
Dose–response curves for carbachol constructed in the presence of the *R. graveolens* extract (15, 30, and 45 μg/mL). Symbols and vertical bars represent means and SEM (n = 4 biological replicates). Data with different superscript symbols differ significantly among groups: p < 0.05

To confirm the antagonist-like effect, dose–response curves of carbachol were constructed in the presence of the plant extract or vehicle medium. Pretreatment with the *R. graveolens* extract at 15, 30, and 45 μg/mL for 20 min produced rightward parallel displacement of the carbachol curves and reduced the maximum contraction to 90.7 ± 3.0 %, 88.7 ± 2.2 %, and 64.5 ± 3.2 %, respectively (Fig. 4).

## Discussion

Stimulation of airway calcium-activated potassium channels induces a sustained increase in the intracytoplasmic calcium concentration in smooth muscle cells [21] and is considered to play a significant role in the pathogenic changes associated with asthma [22]. Meanwhile, carbachol induces contraction of the smooth muscle cells by releasing sarcoplasmic calcium, followed by rapid entry of calcium to the cells [23, 24], thereby stimulating 1,4,5-inositol trisphosphate receptors (IP3Rs) and ryanodine receptors (RyRs) [25], both of which are involved in asthma pathophysiology [26]. In the present study, the *R. graveolens* plant extract was capable of significantly reducing the smooth muscle tension of rat tracheal rings subjected to contraction by a cholinergic agonist (carbachol) and a depolarizing agent (KCl) (Figs. 2 and 3, respectively). Additionally, our previous report demonstrated the presence of cytotoxic effects with high concentrations only of the *R. graveolens* extract (above 400 μg/mL) using an endothelial cellular model [27]. Similarly, previous studies have demonstrated myorelaxant effects of plants from the *Rutaceae* family [28, 29]. For example, it has been indicated that a hydroalcoholic extract of *Ruta chalepensis* decreased KCl-induced contraction of the rat ileum, probably by increasing the production of nitric oxide (NO) and cyclic guanosine monophosphate (GMPc) [30]. Other authors have described that benzofurans, acridinons, psoralens, and other coumarins present in plants from the *Rutaceae* family are able to block potassium currents [31, 32].

Pharmacologically, a competitive antagonist blocks the chain of reactions produced by an agonist, as the antagonist acts on a different site in the receptor within the effector system [33]. Thus, dose–response curves for carbachol (0.1–100 μM) were constructed in the presence of the *R. graveolens* extract (5, 10, and 35 μg/mL) (Fig. 4). The obtained data suggested that our plant extract acted as a non-competitive antagonist, because as the dose of *R. graveolens* increased, the maximum effect exerted by the cholinergic agonist gradually decreased and produced rightward parallel displacement of the carbachol curves.

Among the chemical components present in the *R. graveolens* extract and likely to be responsible for its proven bioactivity are alkaloids, rutin [34], furanocoumarins (psoralen, xanthotoxin, bergapten) [35], acridone epoxides, acridone glucosides, gravacridondiol, and the greatest alkaloid in *R. graveolens* root, rutacridone [36–39]. Within the active principles described in plants, rutin, a natural flavonoid present in many herb families (*Polygonaceae, Rutaceae*, and *Violaceae*), has shown dose-dependent relaxant effects in the rat duodenum [40] and can decrease lipopolysaccharide-induced NO synthesis *in vivo* [41]. It has been established that a methanolic extract of *R. graveolens* contains approximately 4 % rutin [42]. Another study considered that coumarin compounds of an aqueous extract of *R. graveolens* were probably capable of blocking ionic currents [43]. Our phytochemical screening by liquid chromatography of a hydroalcoholic extract of *R. graveolens* revealed the presence of flavonoids (quercetin and rutin) and a furanocoumarin (psoralen), which are probably responsible for the bioactive effects revealed here.

## Conclusions

In conclusion, the main contributions of the present study are the first description of the relaxant effects of *R. graveolens* on rat tracheal smooth muscle and the correlation with its popular use in respiratory diseases. The trachea-relaxant effect can be explained through a non-competitive antagonistic mechanism, possibly involving the blockade of ionic currents. However, more detailed studies are needed to identify the target of the inhibition, and to determine more precisely the pharmacological mechanisms involved in the observed biological effects.

## Methods

### Animals

All the animals used in the procedures in this study were treated in accordance with international principles and local regulations concerning the care and ethical use of laboratory animals. The experimental protocol was approved by the Bioethical Commission of the University of La Frontera, presented to the Bioethical National Committee in CONICYT, and revised by the FONDEF Committee. Sprague–Dawley rats weighing 200–250 g were used in the experiments and purchased from the Animal Breeding Laboratory of the University of La Frontera. The animals were maintained under controlled environmental conditions, with room temperature at 18–22 °C, an alternating 12-h/12-h light/dark cycle, and *ad libitum* water, and feeding with a standardized pellet.

### Drugs and chemicals

Carbachol, anhydrous aminophylline, and KCl were acquired from Sigma-Aldrich (USA). The final concentration of KCl (80 mM) was selected on the basis of previous studies [44, 45].

### Extract preparation

Leaves and aerial parts of *R. graveolens* were collected at Temuco in Southern Chile (38° 44′ 52″ S; 72° 37′ 3″ W; 200 m above sea level). The material was identified by Professor Fernando Romero, Faculty of Medicine, University of La Frontera.

For the purpose of organic extraction, the plant extract was prepared by washing *R. graveolens* leaves with deionized water (Simplicity 185; Millipore, Germany), and drying at 37 °C. The dried leaves were then pulverized, extracted with ethanol/water (4:1) for 3 days, filtered in a vacuum, concentrated in a rotary evaporator, and subjected to lyophilization at −80 °C for 2 days (Chris Alpha1-2; Osterade, Germany) to obtain a viscous mass of dark green extract. Just prior to biological testing, the extract was dissolved in vehicle solution (ethanol 0.1 %, cremophor 0.1 %, dimethylsulfoxide 0.1 %, n-hexanol 0.1 %) to prepare a stock solution of 10 mg/mL.

### Phytochemical screening

The hydroalcoholic freeze-dried *R. graveolens* extract was subjected to phytochemical screening to detect the presence of alkaloids. Briefly, 100 mg of the sample *(R. graveolens* extract) was dissolved in 4 mL of methanol, from which a 150-μL aliquot was taken for examination on a LC-MS MS system, consisting of a liquid chromatograph (Shimadzu, Japan) connected to MDS Sciex Mass Spectrometer QTRAP 3200 (Applied Biosystems, USA), equipped with an electrospray ionization (ESI) source Turbo V™ (AB Sciex, Singapore) at 450 °C. Chromatographic separation was performed with a RP-C18 Column Inertsil ODS-3 (2.1 × 150 mm, 3 mm) (GL Sciences, USA) using an injection volume of 10 μL, a flow rate of 0.2 mL/min, and a column temperature of 35 °C. To separate the standards and samples, a gradient consisting of solvent A (water/acetic acid, 99.9:0.1, v/v), and solvent B (methanol) was applied, followed by a 5-min equilibration between each sample. Data acquisition was performed using the software Analyst 1.5.1 (Applied Biosystems, USA). The ESI parameters were as follows: Cur gas: 137.9 kPa; CAD gas: medium; Gas1: 60 psi; Gas2: 30 psi; capillary voltage: 3500 V. For analysis of samples, the Multiple Reaction Monitoring method was used with three compounds in positive polarity and two transitions for each. The retention times of the standards and their transition precursor ions/fragment ions were considered as the positive identification parameters.

### Preparation of isolated trachea and tension measurement

The animals were euthanized by cervical dislocation and their tracheas were removed. The isolated tracheas were dissected, and connective and adipose tissue adhesions were removed. A tracheal segment was cut into transverse rings of 3–5 mm, followed by connection of the lower and upper extremes to a isometric force transducer LabChart pro 6.1 (ADInstruments, CO, USA) in an isolated organ bath with modified Tyrode’s solution (in mM: NaCl 137; KCl 5.4; CaCl_2_.2H_2_O 2.7; MgCl_2_.6H_2_O 0.5; NaHCO_3_ 11.9; NaH_2_PO_4_.H_2_O 0.45) containing 5.55 mM glucose monohydrate. The solution was maintained at 37 °C and pH 7.3, with constant bubbling of 5 % CO_2_ and 95 % O_2_. The tension was continuously measured by the force transducer. To reach spontaneous equilibration, a tension of 1 g was applied initially for 30 min [46]. To verify the integrity of the tracheal tissue, 80 mM KCl was added initially during each experiment, and only responsive tracheal rings were included in the study.

### Determination of EC_50_ of carbachol

To determine the EC_50_ of carbachol, dose–response curves were recorded using increasing concentrations of the reagent (0.1–100 μM) starting with the basal tension of the rings at approximately 1 g. Changes in the force transducer were analyzed as the percentage of change in tension from the baseline (1 g) to the peak of each dose, with approximately 15-min intervals between each treatment.

### Determination of R. graveolens effects on contractile activity

The trachea-relaxant effects of increasing concentrations (C1, 5 μg/mL; C2, 15 μg/mL; C3, 30 μg/mL; C4, 45 μg/mL) of the hydroalcoholic extract of *R. graveolens* versus those of anhydrous aminophylline (C1, 0.2 mM; C2, 0.4 mM; C3, 0.8 mM; C4, 1.0 mM), as one of the main drugs used to prevent and treat asthma, chronic bronchitis, and other lung diseases [47], were examined. Vehicle (25 μL) was used as a negative control. An increase in tone (contraction) was induced with carbachol (1 μM; 80 % maximum response obtained) or KCl (80 mM). In each experiment, the effects of the four increasing concentrations of the extract, aminophylline, or vehicle on the contracted tracheal smooth muscle were measured after exposing the tracheal segment to each concentration of the solution for 15 min. A decrease in tone was considered to be a relaxant effect, and expressed as a positive percentage change in proportion to the maximum contraction.

With the purpose of establishing the possible antagonistic effect of the plant extract, the rings were pre-incubated with 15, 30, and 45 μg/mL of *R. graveolens* extract for at least 20 min [48] and then induced to contract by adding increasing concentrations of carbachol (0.1, 1.0, 10, and 100 μM).

### Statistical analysis

The data for the different functional parameters evaluated were expressed as means ± SEM. Differences between the groups were analyzed by one-way analysis of variance (ANOVA) followed by Tukey multiple comparison tests. Values of p < 0.05 were considered to be significant. For the tension analysis, LabChart5.0 was used, with data processing by Origin 6 for the dose–response curves.
